# Decoupling Stable Cyclic Deformation From Defect Accumulation in Metals

**DOI:** 10.1002/advs.78331

**Published:** 2026-10-15

**Authors:** Chenjun Ye, Shaofan Ge, Tian Yang, Leyi Tang, Kang Wang, Y. Morris Wang, Jun Ding, Zan Li, Di Zhang, En (Evan) Ma

**Affiliations:** ^1^ State Key Laboratory of Metal Matrix Composites Shanghai Jiao Tong University Shanghai China; ^2^ Department of Materials Science and Engineering University of California Los Angeles California USA; ^3^ Center for Alloy Innovation and Design State Key Laboratory for Mechanical Behaviour of Materials Xi'an Jiaotong University Xi'an China

**Keywords:** amorphous nanoparticles, dislocation absorption, fatigue resistance, laser powder bed fusion, reversible shear transformations

## Abstract

Fatigue resistance in metals is fundamentally constrained by a long‐standing dilemma: the strain hardening required to suppress deformation localization is typically achieved through the accumulation of dislocations and other defects that ultimately promote fatigue damage. Whether cyclic plasticity can be sustained without progressive defect storage remains an unresolved question. Here we demonstrate that this limitation can be overcome through the introduction of amorphous nanoparticles into a crystalline metal matrix. We uncover a cyclic deformation regime characterized by sustained resistance to strain localization yet minimal net defect accumulation over prolonged loading. During cyclic deformation, the amorphous nanoparticles function as efficient dislocation sinks, continuously mitigating defect accumulation in the metallic matrix, while their elastic deformation via reversible shear transformations provides apparent strain hardening of the overall material. These synergistic mechanisms effectively preserve the microstructural integrity of both the metallic matrix and the amorphous nanoparticles over millions of loading cycles, leading to a fatigue life of more than 10^5^ cycles at a fatigue ratio of 0.95, and a ratcheting rate one to three orders of magnitude lower than all known metals and alloys.

## Graphical abstract

Introducing amorphous nanoparticles into a metal matrix enables a cyclic deformation regime where strain hardening and defect accumulation are decoupled. These nanoparticles act as dislocation sinks and undergo reversible shear transformations, sustaining microstructural stability over millions of cycles. The resulting composite achieves exceptional fatigue and ratcheting resistance, with a fatigue ratio of 0.95 and ratcheting rates one to three orders of magnitude lower than known metals.

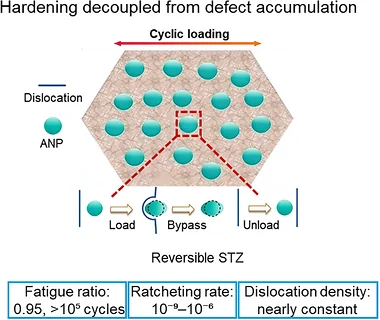

## 1 Introduction

Fatigue failure originates from the progressive accumulation of deformation‐induced defects during cyclic loading and remains one of the most pervasive limitations to the reliability of structural metals [1, 2, 3]. Although cyclic plasticity is often accommodated through the generation and multiplication of dislocations, the resulting defect accumulation promotes strain localization, persistent slip band formation, and ultimately fatigue failure [4, 5, 6, 7, 8]. A long‐standing challenge therefore arises from the dual role of defects during cyclic deformation. On one hand, dislocations are indispensable carriers of plasticity and strain hardening, which suppress localized deformation and delay crack nucleation. On the other hand, the progressive storage of these defects provides the microstructural foundation for fatigue damage. Consequently, cyclic deformation in crystalline metals is generally accompanied by an inherent coupling between hardening and defect accumulation, creating a fundamental limitation in the pursuit of superior fatigue resistance.

Considerable efforts have been devoted to mitigating this limitation through microstructural engineering. Nanotwinned metals exploit coherent twin boundaries to impede dislocation motion while enhancing deformation reversibility [9, 10, 11]. Gradient structures redistribute cyclic strain over multiple length scales and suppress surface strain localization, leading to remarkable improvements in fatigue performance [12, 13, 14]. Precipitates and nanoscale dispersoids stabilize dislocation configurations and inhibit the development of persistent slip bands [15, 16]. These approaches have produced substantial advances in fatigue‐resistant materials, including copper alloys, steels, and other structural metals [17, 18]. Nevertheless, they predominantly operate by enhancing strain hardening through the storage of increasing numbers of defects. As a result, the fundamental coupling between hardening and defect accumulation remains largely intact. This limitation becomes particularly pronounced under asymmetric cyclic loading conditions, such as tension–tension fatigue, where the accumulation of ratcheting strain further accelerates defect storage and damage evolution [19, 20, 21].

An intriguing yet largely unexplored possibility is whether cyclic plasticity can be sustained without substantial net defect accumulation. Achieving such a deformation regime requires a mechanism that accommodates plastic strain while preventing the progressive storage of fatigue‐critical defects. In crystalline materials, however, such behavior is difficult to realize because the mechanisms responsible for stable cyclic deformation are themselves intrinsically associated with defect generation and retention.

Here we demonstrate that amorphous nanoparticles embedded within a crystalline metal matrix provide a route to overcoming this long‐standing limitation. Using laser additive manufacturing, we introduce a high density of intragranular amorphous nanoparticles into a copper matrix and uncover a cyclic deformation regime characterized by exceptional microstructural stability and minimal net defect accumulation during cyclic loading. We show that the amorphous nanoparticles fundamentally alter the interaction between plastic deformation and defect evolution, enabling sustained cyclic deformation while suppressing fatigue‐critical defect storage. As a consequence, the material exhibits extraordinary resistance to both fatigue failure and ratcheting deformation. These findings demonstrate that stable cyclic deformation and defect accumulation need not be intrinsically coupled, challenging the paradigm in the fatigue of crystalline metals and establishing amorphous nanoparticles as a new class of functional reinforcements for fatigue‐resistant structural materials.

## 2 Results and Discussion

### 2.1 Microstructures and Extraordinary Fatigue Resistance

We have developed an in situ fragmentation method that enables the spontaneous refinement of reinforcing particles into amorphous nanoparticles via laser powder‐bed fusion (L‐PBF), by exploiting the high melt temperatures and rapid solidification rate. Dispersoids, initially present as particles or agglomerates, are transiently melted, disintegrated, or rendered interfacially unstable under the coupled thermo‐mechanical and melt‐flow conditions induced by laser irradiation [22]. This in situ nano‐fragmentation is followed by ultrafast melt‐quenching, such that the fragments are re‐dispersed and kinetically trapped within the metallic matrix as uniformly widespread amorphous nanoparticles; such a microstructure has so far been difficult or impossible to access via traditional processing routes [22]. As a model system, we employ high‐purity copper‐based nanocomposites reinforced with boron carbide (B_4_C) ceramics; the latter is chosen for their thermal stability and minimal chemical decomposition or interfacial reaction with the Cu matrix during laser melting. This yields a dense population of intragranular, amorphous B_4_C nanoparticles.

The three‐dimensional metallographic reconstruction reveals a uniform dispersion of nanoscale B_4_C particles throughout the Cu matrix, in the L‐PBF‐fabricated Cu–B_4_C nanocomposites (4 vol% B_4_C), with no detectable cracks or voids in the as‐built state (Figure S1a). Statistical analysis based on scanning electron microscopy (SEM) yields an average B_4_C particle size of 43.5 ± 2.0 nm (Figure S1b). Bright‐field transmission electron microscopy (BF‐TEM) images (Figure 1a) show that the nanoparticles are predominantly located in the interior of Cu grains, where dislocations are also observed as a result of the thermal‐mechanical coupling during L‐PBF processing cycles. Cross‐sectional high‐angle annular dark‐field scanning transmission electron microscopy (HAADF‐STEM) further reveals that the particles are amorphous (Figure 1b). Notably, the Cu–B_4_C interfaces appear clean and largely free of dislocation pileups, despite the substantial mismatch in the coefficients of thermal expansion between Cu (16.5 × 10^−6^ K^−1^) and B_4_C (5.73 × 10^−6^ K^−1^). Energy‐dispersive X‐ray spectroscopy (EDS) mapping (Figure S2a) confirms that the amorphous nanoparticles have an atomic B/C ratio of ∼4:1, while near‐edge X‐ray absorption fine‐structure (NEXAFS) spectroscopy reveals the characteristic local B–C bonding environment of B_4_C (Figure S2b). These results demonstrate that the L‐PBF process enables the formation of metallic materials with uniformly distributed intragranular amorphous nanodispersions.

**FIGURE 1:**
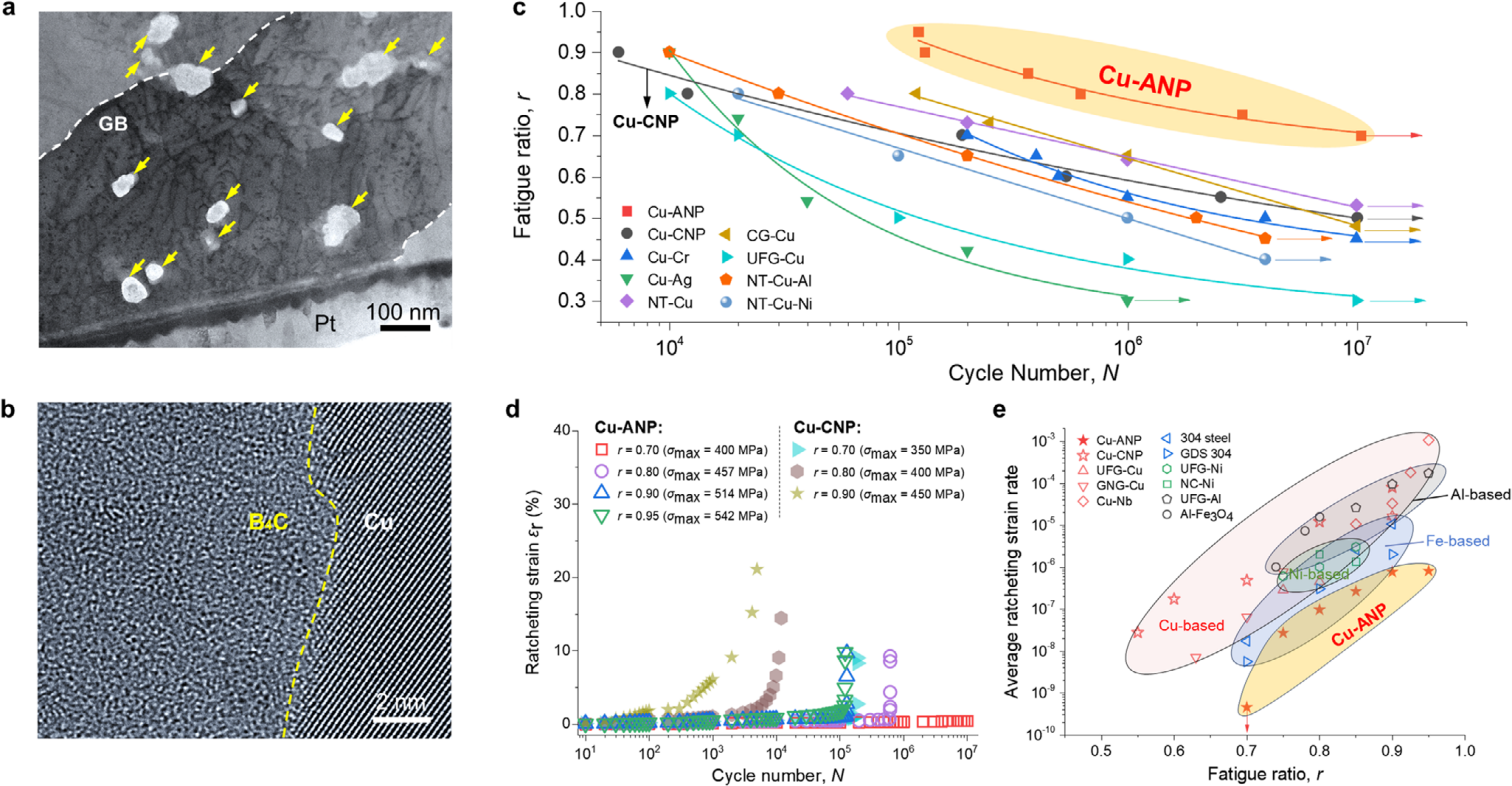
Microstructure and cyclic deformation behavior of Cu–ANP nanocomposites. (a) Bright‐field TEM image showing intragranularly dispersed amorphous nanoparticles (indicated by the yellow arrows) in the Cu matrix, with dislocations visible from thermal cycling. (b) Cross‐sectional HAADF‐STEM image confirming the amorphous nature of the nanoparticles. (c) Fatigue ratio, *r* (σ_max_/σ_UTS_), versus number of cycles to failure (*N*), comparing Cu–ANP with other copper‐based materials. Note that all the referential data and Cu–ANP possess the same stress ratio (*R* = *σ*_min_/*σ*_max_) of 0.1 during the tension‐tension cyclic deformation. Sources of the references are cited in Table S1. (d) Cumulative ratcheting strain as a function of cycle number under tension–tension loading, showing suppressed ratcheting in Cu–ANP compared with its crystallized counterpart (Cu–CNP). (e) Comparison of average ratcheting strain rate versus fatigue ratio for various metallic materials. Sources of the references are cited in Table S2.

We characterized both the uniaxial tensile and cyclic deformation behavior of the L‐PBF Cu–B_4_C nanocomposite containing amorphous nanoparticles (hereafter termed Cu–ANP) at ambient temperature. Quasistatic tensile tests show that the incorporation of only 4 vol.% B_4_C amorphous nanoparticles elevates the ultimate tensile strength (*σ*_UTS_) to 552 ± 5 MPa, representing an ∼80% increase compared with L‐PBF pure copper (310 ± 5 MPa) (Figure S3). To evaluate cyclic deformation and ratcheting behavior, asymmetrical tension–tension cyclic loading was performed under stress control with a stress ratio (*σ*_min_/*σ*_max_) of 0.1. Figure 1c plots the fatigue ratio (*r* = *σ*_max_/*σ*_UTS_) as a function of the number of cycles to failure (*N*_f_) (SI Appendix, Table S1 for reference details). Remarkably, the L‐PBF Cu–ANP nanocomposite exhibits the highest fatigue ratio reported to date among copper‐based materials strengthened by conventional strategies such as alloying, grain refinement, or heterogeneous microstructures under comparable testing conditions. It sustains 10^5^ cycles without failure even at a fatigue ratio of 0.95, a performance unmatched by previous fatigue‐resistant copper architectures, including nanograined [23], gradient [24, 25], and nanotwinned copper [26, 27], at comparable lifetimes.

The nanocomposites also exhibit superior resistance to cyclic ratcheting deformation (Figure 1d) under maximum stresses (*σ*_max_) ranging from 386 to 524 MPa, corresponding to a fatigue ratio (*r*) of 0.7 to 0.95. Figure 1e compares the combinations of average ratcheting rate and fatigue ratio for a variety of metallic materials with different compositions and microstructures (SI Appendix, Table S2 for reference details). As shown, the average ratcheting strain rate of metals/alloys typically ranges from 10^−8^ to 10^−3^ and increases with fatigue ratio. Our nanocomposites exhibit exceptionally low average ratcheting strain rates of 10^−9^ to 10^−6^, which are one to three orders of magnitude lower.

The critical role of the amorphous structure in sustaining the exceptional fatigue resistance is clearly seen in the following comparison. Thermal annealing at 1173 K for 6 h induces crystallization of the amorphous B_4_C nanodispersions (Figure S4), while leaving the grain size of the Cu matrix largely unchanged. As shown in Figure 1c, the resulting Cu–crystallized nanoparticle composite (Cu–CNP) exhibits markedly reduced fatigue resistance, showing a fatigue life one to two orders of magnitude shorter than those of the Cu–ANP counterpart under identical fatigue ratios of 0.7–0.9. The cumulative ratcheting strain in Cu–CNP increases much more rapidly than in Cu–ANP, resulting in ratcheting‐to‐failure lives that are two to three orders of magnitude shorter (Figure 1d).

### 2.2 Synergistic Interplay Between the Matrix and ANPs

We first conducted in situ synchrotron X‐ray diffraction experiments during cyclic loading to uncover the mechanisms responsible for the extraordinary resistance to fatigue damage accumulation and cyclic ratcheting deformation of Cu‐ANP. Asymmetrical tension–tension cyclic loading was performed under stress control with the same stress ratio (*σ*_min_/*σ*_max_) of 0.1 at a fatigue ratio (*σ*_max_/*σ*_UTS_) of 0.8. The high‐energy X‐rays enable real‐time tracking of lattice strain and diffraction peak intensity in Cu–ANP during cyclic deformation (see Methods). Quantitative structural information at different deformation cycles was extracted from the diffraction patterns, as shown in Figure 2a. Figure 2b shows the evolution of the full width at half maximum (FWHM) of the Cu (111), (200), (220), (311), (222), and (400) diffraction peaks as a function of cycle number at the minimum stress. Only minor deviations from the initial FWHM values (on the order of 10^−3^) are observed as cyclic deformation proceeds. By applying the Wilkens model assuming a random distribution of screw dislocations (SI Appendix), the dislocation density was estimated to increase from 7.81 × 10^14^ m^−2^ after 100 cycles to only 9.36 × 10^14^ m^−2^ after 5 × 10^4^ cycles, indicating minimal increase in dislocation density during cyclic loading. Notably, the cumulative plastic strain in Cu–ANP has reached an exceptionally large value (∼409) after a long ratcheting life of 5 × 10^4^ cycles. This absence of dislocation accumulation suggests that the deformation‐induced defects must have been continuously annihilated or recovered under cyclic loading.

**FIGURE 2:**
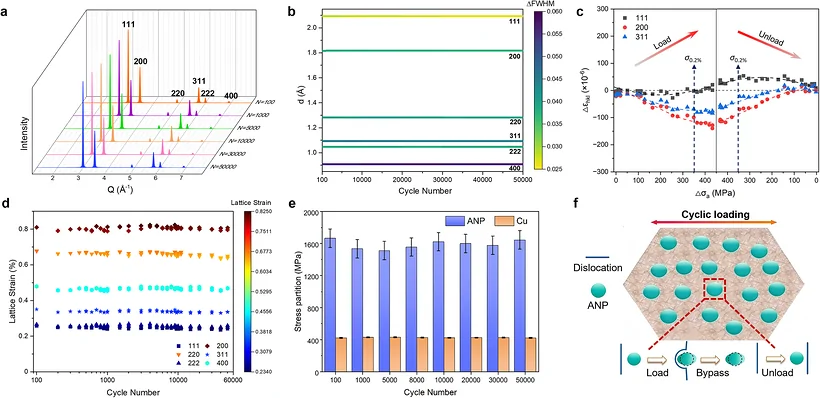
In situ synchrotron X‐ray diffraction analysis of Cu–ANP during cyclic loading. (a) Evolution of diffraction patterns during tension–tension cycling at a fatigue ratio of 0.8. (b) Full width at half maximum (FWHM) of Cu diffraction peaks as a function of cycle number, showing minimal changes throughout cycling (from *N* = 100 to *N* = 50000). (c) Lattice strain divergence as a function of applied stress during a single cycle, revealing early activation of microplasticity and reversible strain behavior. Δσ_a_ is defined as (*σ* – *σ*_min_), where *σ* and *σ*_min_ represent the applied stress and the minimum stress upon cyclic deformation, respectively. (d) Lattice strain at peak stress for different cycles, indicating stable strain partitioning. (e) Stress partitioning between the Cu and ANPs, derived from Cu (311) peak shifts, showing nearly constant stress distribution in both Cu and ANPs without hardening. (f) Schematic illustration of the microstructure stability mechanism during cyclic deformation of Cu‐ANP.

We analyzed lattice strain divergence to quantify microplasticity behavior and stress partitioning during cyclic deformation. Plastic deformation in coarse‐grained (∼1.1 µm for Cu‐ANP) Cu matrix is governed by dislocation‐mediated processes, which produce stress partitioning among different crystallographic planes during loading and unloading. The elastic lattice strain divergence is defined as Δ*ɛ^hkl^*  = (*d^hkl^* – *d*_min_ *^hkl^*)/*d*_min_ *^hkl^* – (*σ* – *σ*_min_)/*E^hkl^*, where *d^hkl^* and *d*_min_ *^hkl^* represent the lattice spacing of the (hkl) plane at an applied stress *σ* and *σ*_min_, respectively, and *E^hkl^* is the corresponding crystallographic elastic modulus. During a single tension–tension cycle, the lattice strain divergence deviates from zero well before the macroscopic yielding point and exhibits nonlinear evolution for different lattice planes of Cu (Figure 2c), revealing the onset of microplastic deformation and stress partitioning during loading. This early activation of microplasticity in Cu can be partly attributed to elastic strain incompatibility between the Cu matrix and the B_4_C nanoparticles. Remarkably, the small peak shifts are fully recovered upon unloading, indicating that the inhomogeneous strain generated during loading is largely reversible. We tracked the lattice strain at the peak stress of different cycles (Figure 2d) and determined the stress partitioning between the Cu matrix and the amorphous nanoparticles using the peak shifts of the Cu (311) reflections as a reference (Figure 2e) [28]. The nearly constant stress carried by both the Cu matrix and the amorphous nanoparticles indicates no significant strain hardening of the Cu matrix during the probed cycles, confirming the absence of progressive dislocation or defect accumulation during cyclic deformation. To assess cyclic stability, we constructed the cyclic stress–strain curve from stable hysteresis loops at different fatigue ratios (Figure S5). The curve is linear (slope ∼1.05 × 10^4^ MPa, R^2^ = 0.993), and fatigue stress–strain curves show constant peak stress over 10^6^ cycles (Figure S6), confirming no cyclic hardening or softening.

The recoverable microstructure after cyclic deformation is consistent with TEM observations: post‐fatigue samples tested at a fatigue ratio of 0.95 reveal neither substantial dislocation storage in the Cu matrix nor pronounced pile‐up around the ANPs (Figure 3a). Atomic‐scale geometric phase analysis (GPA) maps show that residual elastic inhomogeneous strain and interfacial dislocation pile‐up are rare after fatigue testing (Figure 3b), underscoring the strong dynamic accommodation capability of the ANPs. High‐resolution EBSD of Cu–ANP after different numbers of cycles (Figure 3c) shows only a minimal increase in geometrically necessary dislocation (GND) density, from 7.40 × 10^14^ m^−2^ at *N* = 2 × 10^4^ to 8.80 × 10^14^ m^−2^ at *N* = 1 × 10^5^. Atomic force microscopy (AFM) scans likewise reveal no macroscopic shear bands or nanocracks on the fatigued surface. The surface height variation, approximately −100 to 100 nm (Figure 3d,e), is at least an order of magnitude smaller than the micrometer‐scale extrusions observed in fatigued coarse‐grained Cu [24], indicating highly uniform plastic flow during cyclic deformation.

**FIGURE 3:**
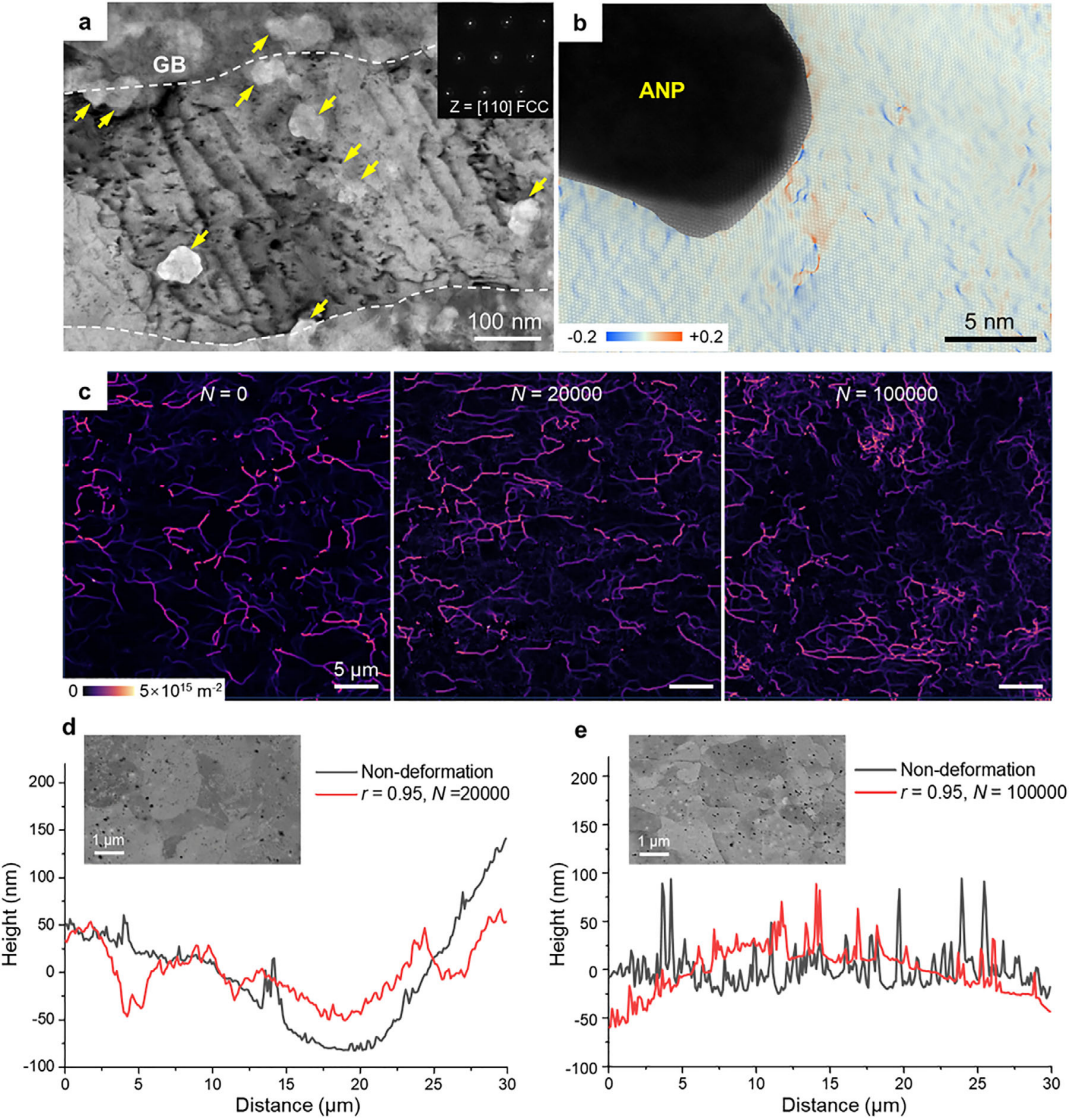
Microstructural stability and deformation features in Cu–ANP after cyclic loading. (a) Bright‐field TEM image of Cu–ANP after fatigue testing at a fatigue ratio of 0.95 and *N* = 10^5^, showing no significant dislocation accumulation or pile‐up nearby ANPs (as indicated by yellow arrows). (b) Atomic‐scale geometric phase analysis (GPA) map, revealing minimal residual strain at the Cu–ANP interface. (c) High‐resolution EBSD showing geometrically necessary dislocation (GND) density evolution with cycling at a fatigue ratio of 0.95. Note that the GND density is 6.19 × 10^14^/m^2^ at *N* = 0, which increases marginally to 7.36 × 10^14^/m^2^ and 8.81 × 10^14^/m^2^ after 20000 and 100000 deformation cycles, respectively. (d, e) Atomic force microscopy (AFM) images to show the height variations of the fatigued surface (fatigue ratio of 0.95), showing minimal surface roughness and absence of shear bands or cracks at two representative cycles (*N* = 20000 and 100000).

With the microscopic information in hand, we are now in a position to explain how the interplay between the matrix and the ANPs leads synergistically to the superb performance above. Conventionally, cyclic deformation in metals containing nanoscale heterogeneities is governed by the progressive accumulation of dislocations and Orowan loops, which generate long‐range internal stresses (back stress) and amplify strain incompatibility, ultimately leading to interfacial stress concentration and fatigue damage [29, 30]. In contrast, the Cu–ANP system reveals a fundamentally different regime in which defect generation and annihilation are dynamically balanced such that they cancel out in each loading‐unloading cycle. This absence of significant dislocation accumulation, together with minimal plastic incompatibility, arises from the interplay between the crystalline Cu matrix and the embedded amorphous B_4_C nanoparticles. By introducing crystal–amorphous interfaces, an alternative strain accommodation mechanism is activated via shear transformations (STs) at the surfaces of the ANPs. That is, imposed strains are accommodated by the excess flexibility (akin to free volume) intrinsic to the ANP amorphous structure, inside which the disordered atomic configurations continuously evolve through bond breaking and reforming. Such collective atomic rearrangements accommodate incoming dislocations and local strain [31], leading to efficient absorption of dislocation loops at the Cu–ANP interfaces while suppressing the build‐up of extra interfacial defect structures. In the absence of accumulating defects, the apparent strain hardening of the composite as a whole is not microstructural in origin, but arises from the increasing load transfer to the reinforcing ANPs, the elastic deformation of which requires higher stress with increasing strain. Note that the majority of this ANP deformation is recovered upon unloading. That is, the vast majority of the STs in the ANPs are actually reversible. Even for those irreversible STs, which are the extreme case, these microplastic events would not develop into any runaway shear band, as the particle is tiny and under the confinement of the surrounding Cu matrix. Cyclic plasticity therefore proceeds with little storage of defects that are not dynamically recovered (Figure 2f). Importantly, the large mismatch in modulus and hence elastic strain field, when the elastic load‐bearing ANPs are coupled with the plastically deforming Cu matrix, gives rise to a high internal back stress (Figure S7). This sustained internal stress promotes backward dislocation motion during unloading and causes reversed plastic deformation, thereby suppressing the accumulation of ratcheting strains.

### 2.3 Atomistic Mechanism of Reversible Shear Transformations and Defect Absorption

To gain atomistic insight into the stable cyclic response of Cu‐ANP, we performed large‐scale molecular dynamics (MD) simulations under stress‐controlled cyclic loading at a fatigue ratio of 0.8, using a representative amorphous nanoparticle diameter of 12 nm. Pre‐existing partial dislocations with associated stacking‐fault regions were introduced into the Cu matrix to directly probe dislocation–ANP interactions. The simulations show that an incoming dislocation is first trapped by the amorphous nanoparticle and then separated into segments that evolve along different pathways. The segment impinging directly on the ANP is absorbed into the amorphous structure, indicating that the ANP acts as an effective sink for dislocations (Figure S8). Meanwhile, the two lateral dislocation segments continue to glide under the applied stress, with their ends moving along the Cu–ANP interface. This bypass process differs fundamentally from the conventional Orowan mechanism around crystalline impenetrable obstacles. In the latter case, dislocations leave behind residual loops that accumulate around the particle after repeated dislocation passage, generating long‐range internal stresses and interfacial stress concentrations. In contrast, after the dislocation bypasses the ANP, no stable dislocation debris remains in the surrounding Cu matrix. Hence, lattice defects propagating through the Cu matrix are not retained as stable interfacial dislocation structures. They are instead absorbed by the ANP and redistributed through local atomic rearrangements, thereby leaving no progressive defect accumulation during cyclic loading and avoiding the stress concentration commonly associated with crystalline particles [32]. We quantified the inelastic strain at the atomic level to elucidate the underlying atomic‐scale processes upon dislocation‐ANP interaction (Figure S9). Pronounced local strain accumulation develops inside the ANPs near the dislocation‐particle intersection sites, corresponding to the activation of abundant STs near the interface of Cu‐ANP. These STs are enabled by local bond switching and positional rearrangement within the ANP [33], and the shear transformation zones (STZs) appear as spatially confined groups of atoms undergoing cooperative non‐affine displacement [34, 35].

Importantly, the STs activated within the ANPs are largely recoverable during cyclic shear loading. Figure 4a reveals the number of atoms within the ANPs with the large nonaffine squared displacement [35], D_min_^2^ > 5 after unloading, as a function of loading cycles. This number increases rapidly during the initial cycles but soon reaches a saturated state, indicating that shear‐transformation activation and recovery become dynamically balanced. This behavior is further resolved within a representative cycle, *N* = 100 (Figure 4b), where the number of ST‐activated atoms increases during loading as the ANPs accommodate incoming dislocations and local strain incompatibility at the crystal–amorphous interface. During unloading, however, this population decreases substantially even though the macroscopic applied stress remains tensile, leading to minimal net accumulation after a complete cycle. The forward and backward rearrangements of representative shear transformations are directly visualized in Figure 4c, confirming that cyclic deformation in Cu–ANP is associated with the repeated and largely reversible rearrangements in the amorphous structure.

**FIGURE 4:**
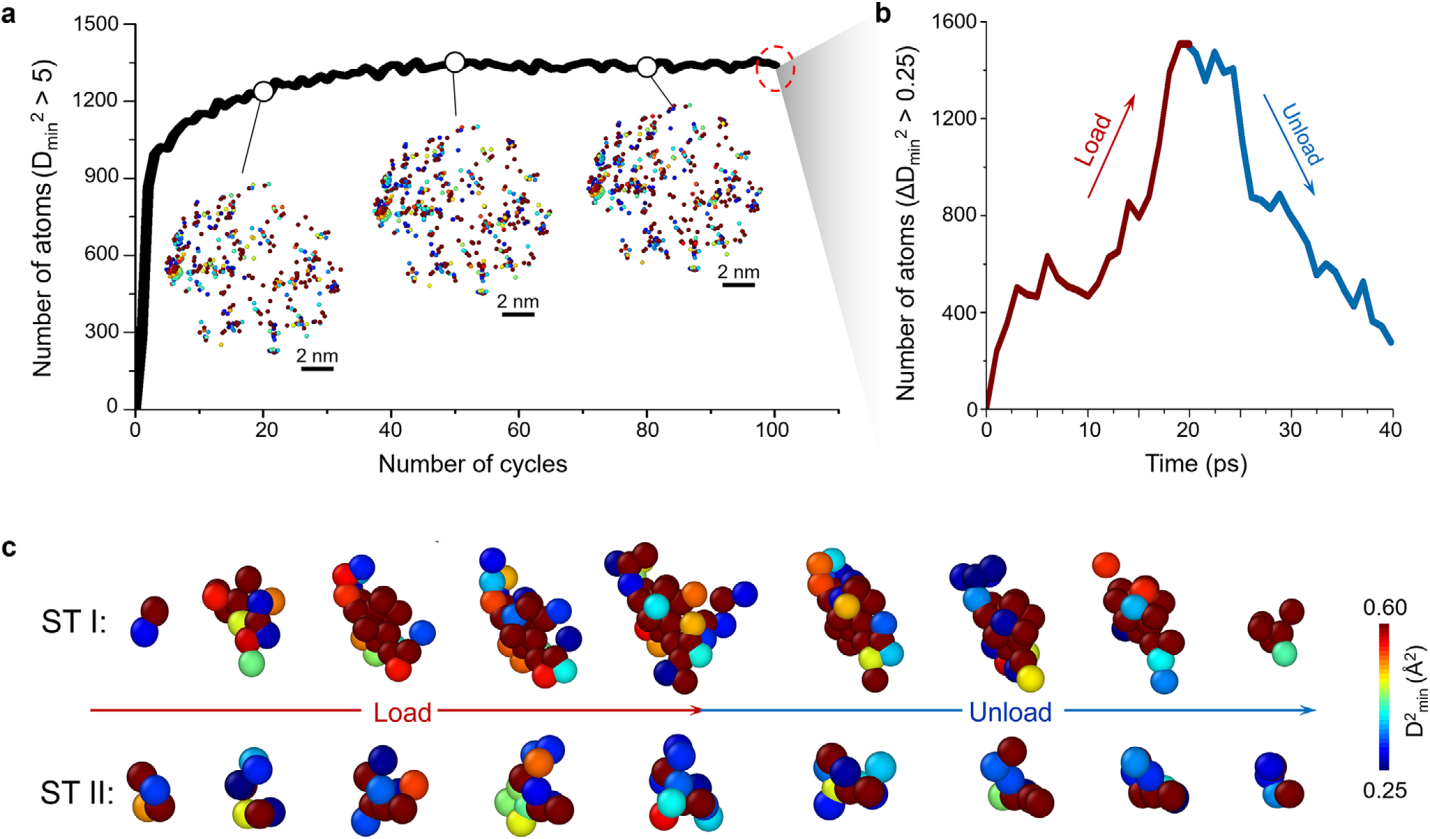
Atomistic mechanisms of cyclic deformation revealed by MD simulations. (a) Evolution of the number of atoms within ANPs exhibiting the nonaffine squared displacement, D_min_^2^, larger than 5, after unloading as a function of loading cycle. Insets show the spatial distribution of the ST‐activated atoms. (b) Variation in the number of atoms with ΔD_min_^2^ > 0.25 within the ANPs during the representative loading‐unloading cycle (*N* = 100), where ΔD_min_^2^ indicates the nonaffine squared displacement relative to the atomic configuration at the beginning of this cycle. (c) Atomic structural evolution of two representative STs expanding during the 100th cycle. Note that the STs are almost recovered after unloading.

The pronounced recovery of STs near the Cu‐ANP interface indicates that their deformation differs from the irreversible shear‐transformation avalanches often associated with shear localization in monolithic metallic glasses. In metallic glasses, ST activation is typically associated with irreversible atomic shuffling and persistent generation of “free volume”. By contrast, the near‐complete restoration of the D_min_^2^ distribution in amorphous B_4_C upon unloading suggests that many interfacial deformation events correspond not to mature irreversible STs, but to reversible or weakly metastable shear‐transformation embryos confined within a covalently bonded amorphous network. Similar reversible local rearrangements have been identified in cyclically deformed amorphous solids below a critical strain amplitude, where atoms repeatedly return to their original configurations after each loading cycle rather than accumulating permanent plasticity [36, 37]. Such reversible local structural excitations are similar to the β‐relaxation‐type configurational fluctuations extensively discussed in amorphous systems [38, 39], but in the present amorphous ceramic particles they are likely further constrained by the directional B–C bonding network.

The reversibility of local STs near the Cu–ANP interface is governed by two coupled factors: the local configurational energy landscape of the amorphous ceramic network and the internal stress field generated by elastic strain incompatibility between the soft Cu matrix and the stiff ANP. The Cu–ANP interface is structurally disordered and relatively loosely packed compared with the crystalline Cu interior, providing local configurational freedom for low‐barrier bond switching and cooperative atomic shuffling. Stress concentration arising from the elastic mismatch between Cu and the ANP further promotes ST activation under modest applied stresses, and the STs in turn relax the stress concentration. During unloading, however, locally reversed shear stress develops near the Cu–ANP interface even while the macroscopic applied stress remains positive (Figure S10 and Movie S1), arising from the elastic deformation incompatibility accumulated during loading. This backward‐directed local stress promotes reverse motion of dislocation segments in the Cu matrix and simultaneously biases atomic rearrangements inside the ANP toward their previous configurations. This behavior resembles reversible transitions between neighboring local minima on the rugged potential‐energy landscape of glasses [40, 41], and can be inferred from the locally fluctuating shear stress nearby STs during unloading (Figure S11).

The robustness of the observed mechanism was further examined using additional simulations at 300 K with a stress ratio τ_min_/τ_max_ = 0.1 and with an enlarged simulation cell. In both cases, limited residual dislocation accumulation at the Cu–ANP interface and recovery of STs within the ANP were retained (Figures S12 and S13). These observations suggest that covalently bonded amorphous network possess a recoverable shear‐transformation regime that has been underappreciated in conventional descriptions of amorphous plasticity. Specifically, the ANPs do not act as damage‐accumulating weak regions, rather they are sinks that dynamically recover lattice defects, as well as a reservoir of local STs that flip back and forth. The resultant defect‐absorption mechanism provides the atomistic basis for the reduced irreversible plastic‐strain accumulation as well as the enhanced ratcheting resistance. The mechanism does not depend on Cu specifically. Any crystalline matrix containing nanoscale amorphous inclusions capable of reversible local rearrangements may exhibit similar behavior. Note that the Cu–ANP interface remains structurally stable against decohesion [42], with calculations demonstrating that interfacial failure does not occur before substantial plastic deformation is activated in the Cu matrix (Figure S14).

## 3 Conclusions

We have identified a cyclic deformation regime in which substantial plastic deformation proceeds with minimal net defect accumulation. This behavior is enabled by amorphous nanoparticles that simultaneously act as sinks for lattice defects and reservoirs for largely reversible shear transformations. Unlike conventional strengthening particles, which promote the storage of dislocations and the buildup of internal damage, amorphous nanoparticles dynamically accommodate strain while maintaining microstructural stability during cyclic loading. The resulting decoupling of stable cyclic deformation from defect accumulation alters the relationship between strengthening and fatigue damage in metallic materials. Exceptional fatigue and ratcheting resistance emerge as direct consequences of this deformation mechanism. More broadly, these findings establish amorphous nanoparticles as a new class of functional reinforcements and provide a general framework for designing structural materials capable of sustaining cyclic deformation while suppressing fatigue‐critical defect storage.

## 4 Materials and Methods

### 4.1 Sample Preparation

High‐purity copper powders (average size of ∼35 µm) were mixed with crystalline B_4_C microparticles (average size of ∼4 µm, 4 vol.%). The powder mixing was facilitated by using a high‐shear dispersion facility (Nobilta, Hosokawa Micron Ltd., Japan) for 20 min at a rotation speed of 1600 rpm under the protection of argon. Porosity‐free bulk composites (density: >99.8%) were then built using a selective laser melting machine (S210, BLT, China) under high‐purity argon to minimize oxidation (oxygen content: < 50 ppm). This system was equipped with a single‐mode fiber laser (500 W, 1064 nm wavelength) with a spot size of 60 µm. A continuous scanning strategy involving a 67‐degree rotation before scanning another layer was adopted to randomize the crystal orientations and alleviate residual stresses in the built samples. The key printing parameters were as follows: laser power of 300 W, scan speed of 800 mm/s, hatch distance of 0.11 mm, and layer thickness of 0.03 mm, yielding an input volumetric energy density of 113.6 J/mm^3^. For comparison, the as‐built copper nanocomposites were annealed at 1173 K for 6 h in a glove box with oxygen and water contents of less than 0.5 ppm to crystallize the B_4_C nanoparticles (termed Cu‐CNP).

### 4.2 Mechanical Testing

Dog‐bone‐shaped specimens for quasistatic uniaxial tensile tests were machined from the as‐built Cu–ANP nanocomposites using electrodischarge machining (EDM). The specimens had a gauge length of 10 mm, a width of 2 mm, and a thickness of 1 mm, with the tensile direction perpendicular to the build direction. All specimens were ground using SiC grit papers from 400# to 4000#, followed by fine polishing with 3 and 1 µm diamond suspensions, and finally electropolished in a solution of perchloric acid (10%) and alcohol (90%) at ambient temperature to obtain a smooth, strain‑free surface (root‑mean‑square roughness R_a_ < 0.1 µm). Quasistatic tensile tests were conducted on a universal testing machine (Instron 3344) at a constant strain rate of 5 × 10^−4 ^s^−1^ at room temperature. Strain was measured using a static axial clip‑on extensometer (Model 2630‑101, Instron) with a gauge length of 6 mm and a displacement resolution of 1 µm. At least three tensile tests were performed for each sample type to ensure statistical reliability.

For fatigue and ratcheting tests, specimens were machined with the same dog‑bone geometry but with a reduced gauge section of 2 mm in length, 1.5 mm in width, and 1 mm in thickness, conforming to the ASTM E466‑21 standard for pull‑pull fatigue testing. The surfaces were prepared following the same grinding, polishing, and electropolishing procedure as used for tensile specimens. Asymmetrical tension‑tension cyclic loading was performed under stress control using a high‑frequency fatigue testing machine (Care M‑6000, Tianjin, China) at a frequency of 15 Hz (sinusoidal waveform). The stress ratio (σ_min_/σ_max_) was kept constant at 0.1. The maximum cyclic stress (σ_max_) was varied from 386 to 524 MPa, corresponding to fatigue ratios (*r* = σ_max_/σ_UTS_) of 0.7 to 0.95. Each fatigue test was stopped either when the specimen completely fractured or when 1 × 10^7^ cycles were reached without failure. The cumulative ratcheting strain was recorded as a function of cycle number, and a two‐dimensional digital image correlation (DIC) was employed to accurately measure cyclic and ratchetting strains.

### 4.3 In Situ Synchrotron X‐Ray Diffraction Testing

In situ synchrotron X‐ray diffraction (SXRD) tests during cyclic loading were conducted at BL12SW of the Shanghai Synchrotron Radiation Facility (SSRF). High‐energy X‐rays with an energy of 88 keV (wavelength λ = 0.1409 Å) were used to enable real‐time tracking of lattice strain, diffraction peak intensity, and peak broadening in the Cu–ANP nanocomposite during fatigue cycling. Dog‐bone‐shaped specimens with nominal gauge dimensions of 2 mm (length) × 1.5 mm (width) × 1 mm (thickness) were machined from the as‐built Cu–ANP samples. The specimens were loaded using a miniature high‐temperature electro‐hydraulic servo fatigue testing machine (Model HD005, Huakong (Suzhou) Intelligent Equipment Co., Ltd.) installed on the beamline. Asymmetrical tension‐tension cyclic loading was performed under stress control with a stress ratio σ_min_/σ_max_ = 0.1 and a frequency of 5 Hz (sinusoidal waveform). The maximum cyclic stress was set to 442 MPa, corresponding to a fatigue ratio σ_max_/σ_UTS_ = 0.8. The diffracted intensity from the sample was collected in transmission geometry using a two‐dimensional (2D) PILATUS3 X CdTe 2M detector. The detector was positioned at a distance of 1021.951 mm from the sample, providing a Q‐space resolution better than 0.01 Å^−1^. A monochromatic X‐ray beam with a spot size of 0.5 mm × 0.2 mm was used to probe the gauge section of the specimen during fatigue testing. Data acquisition was performed at specific intervals during the cyclic loading: for the first 1000 cycles, diffraction patterns were collected every 100 cycles; thereafter, patterns were collected every 1000 cycles until the 50 000th cycle. At each time point, 100 individual frames were recorded with a total collection time of 5 ms per frame (consisting of a 4 ms acquisition time and a 1 ms readout/exposure time). The accumulated frames were summed to improve the signal‐to‐noise ratio. Data processing was performed using GSAS‐II software.

### 4.4 Microstructure Characterization

Electron backscatter diffraction (EBSD) was performed using a high‐resolution EBSD system (Symmetry S2, Oxford Instruments) equipped on a field‐emission scanning electron microscope (SEM, Scios 2, Thermo Fisher Scientific). An accelerating voltage of 30 kV, a beam current of 13 nA, and a step size of 50 nm were used for EBSD data acquisition. Grain boundaries with misorientation angles of 2°–10° and >10° were considered as low‐angle grain boundaries (LAGBs) and high‐angle grain boundaries (HAGBs), respectively. The kernel average misorientation (KAM) was calculated with a second‐nearest‐neighbor kernel to map the local orientation gradients and to estimate the geometrically necessary dislocation (GND) density according to the relationship *ρ*_GND​_ = 2*θ*/(*ub*), where *θ* is the local misorientation (in radians), *u* is the step size, and *b* is the Burgers vector. Transmission electron microscopy (TEM) and scanning transmission electron microscopy (STEM) were conducted on a Spectra Ultra microscope (Thermo Fisher Scientific) equipped with a double spherical aberration corrector for both probe‐forming and image‐forming lenses, operating at an accelerating voltage of 300 kV. The microscope was also fitted with a Super‐X energy‐dispersive X‐ray spectroscopy (EDS) system (four silicon drift detectors) for compositional mapping. TEM specimens were prepared using a focused ion beam (FIB) system (Helios 5 UX, Thermo Fisher Scientific) with a standard lift‐out procedure. A final low‐energy milling step (5 kV, 27 pA) was applied to remove the Ga‐contaminated surface layer. To further reduce beam‐induced amorphous damage, the specimens were subjected to low‐voltage Ar^+^ ion milling (Nanomill 1040, Fischione) at 800 V for 10 min. High‐angle annular dark‐field (HAADF) STEM imaging was performed with a convergence angle of 25 mrad and collection semi‐angles of 50–180 mrad, providing atomic‐number (Z) contrast. The amorphous nature of the B_4_C nanoparticles was confirmed by the absence of lattice fringes in HRTEM images and by the diffuse halos in selected‐area electron diffraction (SAED) patterns.

### 4.5 Molecular Dynamics Simulations

Molecular dynamics (MD) simulations were performed using the Large‐scale Atomic/Molecular Massively Parallel Simulator (LAMMPS) [43] software package to investigate the interactions between dislocations and ANP (B_4_C amorphous nanoparticle) during the periodic reciprocating motion of a copper nanocomposite. The interatomic interactions were modeled using the following potentials: the embedded atom method potential for Cu–Cu interactions [44]; the Stillinger–Weber (SW) potential for B–B, C–C, and B–C interactions within the nanoparticles [45]; and the Lennard‐Jones potential for the interfacial Cu–B [46, 47] and Cu–C [33] interactions. The simulation cell, oriented with the X, Y, and Z axes aligned along the [10$\bar{1}$], [1$\bar{2}$1], and [111] crystallographic directions of the Cu matrix, respectively, has dimensions of 38.2 × 22.1 × 25.0 nm with pre‐existing edge dislocations of $b\;=a_{0}\;/2\;\left[10\bar{1}\right]$ on the {111} plane. ANP with a radius of 6 nm was constructed by assembling B_11_C clusters and C–B–C chains [45], followed by energy minimization using the SW potential. The accuracy of the above ANP generation method has been validated in our previous work [22]. Boundary conditions were set as pps in LAMMPS notation: periodic in X and Y, and shrink‑wrapped (non‑periodic) in Z. The entire system contains approximately 1.75 million atoms and was first optimized using the conjugate gradient algorithm. The deformation simulation was conducted under an isothermal‐isobaric (NPT) ensemble, and the time step in the simulation was 2 fs. After system optimization, the temperature was maintained at 10 K, and the pressure was controlled at zero for an equilibration run of 30 000 steps. In order to simulate fatigue testing under stress control, the top and bottom regions, each with a thickness of 5 atomic layers, are selected as loading layers. These two regions are driven in a reciprocating motion along the *X* direction, with the direction periodically reversed. The absolute value of velocity is controlled such that the overall absolute shear strain rate of the model is 10^9^ s^−1^. A closed‐loop stress‐control scheme was used to cycle the shear stress between 0 and 1 GPa with a triangular waveform for 100 cycles. The peak shear stress of 1 GPa was selected with reference to the monotonic shear response of the same model, where additional interfacial dislocations begin to form at approximately 1.25 GPa, corresponding to an MD fatigue ratio of about 0.8. To examine the sensitivity of the MD results to temperature, stress ratio, particle structure, and model size, three additional simulations were also performed. The Cu–ANP model was simulated at 300 K with a stress ratio τ_min_/τ_max_ = 0.1 for 100 loading‐unloading cycles. The τ_*max*_ =  1.0 GPa remain unchanged. An enlarged Cu–ANP model with dimensions of approximately 38.2 × 33.2 × 37.6 nm and the same 6 nm particle radius was subjected to 10 loading–unloading cycles under 0–0.5 GPa shear stress to assess finite‐size and boundary effects. In addition, a Cu–CNP model containing a crystalline B_4_C nanoparticle of the same size was constructed [48]. The stable crystalline B_4_C structure was orthogonalized, expanded, annealed at 300 K, and energy minimized before being embedded into the Cu matrix. The Cu–CNP model was subjected to the same cyclic shear loading as the original Cu–ANP model. The nonaffine squared displacement (D_min_^2^) was calculated using OVITO software [49], where the cutoff radius was set to be 0.5 nm to encompass two nearest neighbor coordination shells. The OVITO software was also adopted to produce the shear atomic strain map and visualize the atomic structures.

## Author Contributions

Z.L. designed the research. Z.L. and D.Z. supervised the project. C.Y., T.Y. and L.T. performed the experiments. S.G., K.W., D.Z. and Y.M.W. analyzed the data. J.D. carried out the MD simulations. Z.L., J.D. and E.M. wrote the paper. All co‐authors contributed to the discussions.

## Conflicts of Interest

The authors declare no conflicts of interest.

## Supporting information




**Supporting File 1**: advs78331‐sup‐0001‐S1.mp4.

**Supporting File 2**: advs78331‐sup‐0002‐SuppMat.docx.

## Data Availability

The data that support the findings of this study are available from the corresponding author upon reasonable request.
