# Supplementary material for: Decoupling Stable Cyclic Deformation From Defect Accumulation in Metals
Source: Adv Sci (Weinh). 2026 Oct 15:e78331. Online ahead of print. doi: 10.1002/advs.78331 (PMC13672251; doi:10.1002/advs.78331)
Supplement: Supplementary file 1 [file ADVS-9999-e78331-s001.docx]

Supporting Information for

**Decoupling stable cyclic deformation from defect accumulation in metals**

Chenjun Ye^1#^ | Shaofan Ge^1#^ | Tian Yang^1^, Leyi Tang^1^ | Kang Wang^1^ | Y. Morris Wang^2^ | Jun Ding^3*^ | Zan Li^1*^ | Di Zhang^1*^ | En (Evan) Ma^3*^

^1^State Key Laboratory of Metal Matrix Composites, Shanghai Jiao Tong University, Shanghai 200240, China | ^2^Department of Materials Science and Engineering, University of California, Los Angeles, CA, USA. | Center for Alloy Innovation and Design, State Key Laboratory for Mechanical Behaviour of Materials, Xi’an Jiaotong University, Xi’an 710049, China

**Correspondence:** Jun Ding **(**[dingsn@xjtu.edu.cn](mailto:dingsn@xjtu.edu.cn)) | Zan Li ([njulizan@sjtu.edu.cn](mailto:njulizan@sjtu.edu.cn)) | Di Zhang ([zhangdi@sjtu.edu.cn](mailto:zhangdi@sjtu.edu.cn)) | En (Evan) Ma ([maen@xjtu.edu.cn](mailto:maen@xjtu.edu.cn))

**This PDF file includes:**

Supporting text

Figures S1 to S14

Table S1 and S2

SI References

**Supporting Information Text**

**The calculation of the dislocation density**

Data processing was performed using GSAS‑II software. The 2D diffraction patterns were integrated over an azimuthal angle range of 82~98° to obtain one‑dimensional intensity versus 2*θ* profiles. A standard LaB_6_ powder sample (NIST SRM 660c) was measured under identical experimental conditions to determine the instrumental resolution function and the instrumental full width at half maximum (FWHM, *β*_inst_) for each reflection. Single‑peak fitting was carried out using a pseudo‑Voigt function (a linear combination of Gaussian and Lorentzian components) to extract the peak position (2*θ*), integrated intensity, and measured FWHM (*β*_meas_). The physical broadening (*β*_phy_) due to microstructural defects was then obtained by deconvoluting the instrumental contribution using the Gaussian approximation:

$\beta_{phy}=\sqrt{\beta_{meas}^{2}-\beta_{inst}^{2}}$	(1)

The lattice strain for the {hkl} reflection, *ε_hkl_*, was calculated by:

$\varepsilon_{hkl}=\frac{d_{hkl}-d_{0, hkl}}{d_{0, hkl}}$	(2)

Where *d_hkl_* and *d_0,hkl_* denote the interplanar spacings of the {hkl} planes under loading and in the stress‑free state, respectively. The full width at half maximum of the Cu diffraction peaks (111), (200), (220), (311), (222), and (400) was extracted as a function of cycle number.

To quantify the evolution of dislocation density during cyclic deformation, the physical broadening was analyzed using the modified Williamson–Hall method [1]. The total physical broadening in reciprocal space, Δ*K*, is given by:

$\Delta K=\frac{0.9}{D}+\left( \frac{\pi A^{2}b^{2}}{2} \right)^{\frac{1}{2}}\rho^{\frac{1}{2}}\left( K\bar{C}^{\frac{1}{2}} \right)$	(3)

where *K* = 2sin*θ*/*λ* is the scattering vector magnitude, *D* is the volume weighted crystallite size, *b* is the magnitude of the Burgers vector (*b* = 0.2556 nm for Cu), *A* is the Wilkens parameter [2] (taken as 2 here, representing a random distribution of screw dislocations), and $\bar{C}$ is the average dislocation contrast factor. For pure copper (FCC), $\bar{C}$ was calculated as

$\bar{C}=\bar{C}_{h00}\left[ 1-q\frac{h^{2}k^{2}+k^{2}l^{2}+l^{2}h^{2}}{\left( h^{2}+k^{2}+l^{2} \right)^{2}} \right]$	(4)

with constants $\bar{C}$_h00_=0.242 and *q*=−0.50 for screw dislocations, derived from the elastic constants of Cu (*C*_11_=170 GPa, *C*_12_=122.5 GPa, *C*_44_=75.8 GPa).

By plotting Δ*K* versus $K\bar{C}^{1/2}$ for the available reflections and performing a linear regression (weighted by the inverse of the variance), the slope, *S*, can be obtained by:

$S=\left( \frac{\pi A^{2}b^{2}}{2} \right)^{\frac{1}{2}}\rho^{\frac{1}{2}}$	(5)

The dislocation density was then calculated as:

$\rho=\frac{2S^{2}}{\pi A^{2}b^{2}}$	(6)

Using this method, the dislocation densities in the Cu–ANP nanocomposite were determined to be 7.81×10^14^ m^−2^ after 100 cycles and 9.36×10^14^ m^−2^ after 5×10^4^ cycles. The lattice strain divergence (Δ*ε^hkl^*) was further calculated to quantify stress partitioning and microplasticity, as described in the main text.

**Figure S1.** Microstructure of the L-PBF-fabricated Cu–B_4_C nanocomposite. (a) Three-dimensional reconstructed scanning electron microscopy (SEM) image of the composite. A cross-sectional electron backscatter diffraction (EBSD) map is superimposed to reveal the equiaxed grain structure. Melt-pool boundary (MPB) and the build direction (BD) are indicated by the yellow dashed lines and white arrow, respectively. (b) Particle size distribution of the amorphous B_4_C nanoparticles measured from SEM images, yielding an average diameter of 43.5 ± 2.0 nm.

**Figure S2.** Chemical and structural characterization of the amorphous nanoparticles (ANPs). (a) Energy-dispersive X-ray spectroscopy (EDS) elemental maps showing enrichment of B and C within the nanoparticles, with an atomic B/C ratio of approximately 4:1. (b) Near-edge X-ray absorption fine-structure (NEXAFS) spectrum at the B K-edge of nanoparticles extracted from the as-built composite, confirming the local B–C bonding environment characteristic of B_4_C.

**Figure S3.** Representative engineering stress–strain curves of the L-PBF Cu–ANP nanocomposite (4 vol.% B_4_C) and L-PBF pure Cu obtained under quasistatic uniaxial tension at a strain rate of 5 × 10^-4^ s⁻¹. The Cu–ANP nanocomposite achieves an ultimate tensile strength of 552 ± 5 MPa, compared with 310 ± 5 MPa for L-PBF pure Cu.

**Figure S4.** Electron backscatter diffraction (EBSD) inverse pole figure (IPF) maps of Cu–ANP after annealing at 1173 K for different durations: (a) 0 h, (b) 3 h, and (c) 6 h. The average grain size of the Cu matrix remains largely unchanged at approximately 1.1 μm.

**Figure S5**. Cyclic stress–strain curve (CSSC) of Cu-ANP. The curve is constructed by connecting the peak points (ε_max_, σ_max_) of stable hysteresis loops obtained at the half-life (Nf/2) for six different fatigue ratios (r=0.70, 0.75, 0.80, 0.85, 0.90, 0.95). The data points follow a linear trend, indicating that the material exhibits no significant cyclic hardening or softening under stress-controlled tension–tension loading. The slope of the linear fit is approximately 1.05×10^4^ MPa.

**Figure S6.** Fatigue stress–strain curves (FSSC) of Cu–ANP at six fatigue ratios. For each fatigue ratio, representative hysteresis loops are shown at selected cycle numbers (e.g., N=10^2^ to 10^6^). The peak stress remains constant throughout cycling for all stress levels, while the loops shift slightly along the strain axis due to ratcheting accumulation. No progressive change in the loop shape or peak stress is observed, confirming cyclic stability and the absence of cyclic hardening or softening.

**Figure S7.** The long-range internal stress (or back stress) calculation for Cu–ANP during cyclic loading. The stress is estimated from one typical loading–unloading hysteresis loops at *r* = 0.8, The thermal component of the flow stress ($\text{σ}^{\text{*}}$) is identified as the initial nonlinear stress relaxation upon unloading [3-4]. Excluding $\text{σ}^{\text{*}}$from the linear elastic segment allows accurate determination of the unloading yield stress ($\text{σ}_{\text{u}}$, 169 MPa) and reloading yield stress ($\text{σ}_{\text{r}}$, 409 MPa). Both $\text{σ}_{\text{u}}$ and $\text{σ}_{\text{r}}$ are composed of the back stress ($\text{σ}_{\text{b}}$) and the effective stress ($\text{σ}_{\text{eff}}$), with $\sigma_{u}=\sigma_{b}-\sigma_{eff}$ and $\sigma_{r}=\sigma_{b}+\sigma_{eff}$. The $\text{σ}_{\text{b}}$ is calculated using the Dickson method [5-6], $\text{ σ}_{\text{b}}\text{=}\left( \text{σ}_{\text{u}}\text{+}\text{σ}_{\text{r}} \right)\text{/2}$, and is stable at 289 MPa from 2×10^4^ to 1×10^5^ cycles, corresponding to ~64% of the maximum applied stress (~442 MPa).

**Figure S8.** Molecular dynamics (MD) simulation sequence of Cu–ANP under cyclic shear loading. The nanoparticles are shown in gray, stacking-fault atoms in red, and dislocation lines identified by Dislocation Extraction Algorithm (DXA) in green. An incoming dislocation is first trapped by the amorphous nanoparticle and subsequently separated into segments that evolve along different pathways. The segment directly impinging on the ANP is absorbed into the amorphous structure, indicating that the ANP serves as an effective dislocation sink and prevents the accumulation of Orowan loops at the interface.

**Figure S9.** Distribution of atomic shear strain in ANPs during dislocation absorption. Dislocation lines identified by DXA in green. Atoms with equivalent atomic strain increments below 0.02 are omitted for clarity. Note that regions within the ANPs where dislocation absorption occurs exhibit significant local strain changes (as indicated by dashed circles).

**Figure S10.** Atomic stress distribution in Cu–ANP during representative loading (a) and unloading (b) at cycle *N* = 100. Dislocation lines identified by DXA in green. Red and blue regions correspond to local shear stresses with positive and negative values, respectively. Locally negative shear stress develops near the Cu–ANP interface even while the applied macroscopic stress remains positive, driving the back-motion of dislocation segments in the Cu matrix. This locally reversed stress arises from elastic strain incompatibility between the soft Cu matrix and the stiff ANPs.

**Figure S11.** MD simulation results showing the local shear stress distribution within the ANP during cyclic deformation of Cu–ANP at cycle *N* = 100. (a) and (b) show the atomic stress distributions at the maximum and minimum applied stresses, respectively. Pronounced stress heterogeneity develops within the ANP, leading to local regions of negative shear stress even when the macroscopic stress remains positive. Such local stress reversal facilitates the recovery of shear transformations during unloading.

**Figure S12.** Atomic configurations showing the dislocation–particle interactions under different simulation conditions. The nanoparticles are shown in gray, stacking-fault atoms in red, and dislocation lines identified by DXA in green. (a) Cu–ANP model after 100 loading–unloading cycles at 300 K under a peak shear stress of 1 GPa and τ_min_/τ_max_ = 0.1. (b) Enlarged Cu–ANP model after 10 loading–unloading cycles with the shear stress cycled between 0 and 0.5 GPa. (c) Cu–crystallized nanoparticle composite (Cu–CNP) model after 10 loading–unloading cycles with the shear stress cycled between 0 and 1 GPa. Panels (a) and (b) show limited residual dislocation accumulation near the particle–matrix interface. Figure (c) shows that significant interfacial dislocations and extended stacking faults were observed in the Cu–CNP model.

**Figure S13.** Atomistic mechanisms of shear transformations (STs) recover revealed by MD simulations. (a) Variation in number of atoms with D_min_^2^ > 70 within the ANP during a representative loading–unloading cycle (N = 100) of the ANP model. D_min_^2^ was calculated relative to the initial configuration before deformation, and the absolute D_min_^2^ values were used for the statistics. (b) Variation in number of atoms with ΔD_min_^2^ > 0.75 within the ANP during a representative loading–unloading cycle (N = 10) of the enlarged Cu–ANP model. ΔD_min_^2^ was evaluated using the same procedure as in the main text, with the configuration at the beginning of the corresponding cycle used as the reference. The number of highly nonaffine atoms decreases as the stress decreases indicating substantial recovery of local atomic rearrangements within the ANP in both models.

**Figure S14.** MD simulation of the deformation of Cu–ANP under continuous monolithic shear. (a) Interaction of dislocations, divided into two partials, with the ANP during shear deformation. (b) Shear stress versus shear strain for the Cu–ANP nanocomposite; the peak stress (dashed arrow) marks the onset of extensive plastic deformation in the Cu matrix, after which the ANP no longer impedes deformation. (c) Atomic structure at the peak stress in (b), with FCC Cu atoms removed to highlight the interface layer (white) on the Cu side. (d) Enlarged view of the selected region in (c), showing the robust Cu–ANP interface.

**Table S1.** Summary of fatigue life and fatigue ratio for copper‑based materials reported in Fig. 1c. For each material, the table provides the fatigue ratio *r* =*σ*_max_/*σ*_UTS_, number of cycles to failure *N_f_*, and the corresponding reference.

| Materials |  |  |  |  |  |  |  | Ref. | Symbol |
| --- | --- | --- | --- | --- | --- | --- | --- | --- | --- |
| Cu-B_4_C ANP | *r* | 0.95 | 0.9 | 0.85 | 0.8 | 0.75 | 0.7 | This work |  |
|  | *N_f_* | 1.22  x10^5^ | 1.30 x10^5^ | 3.67 x10^5^ | 6.22 x10^5^ | 3.15 x10^6^ | 1.04 x10^7^ |  |  |
| Cu-B_4_C CNP | *r* | 0.9 | 0.8 | 0.7 | 0.6 | 0.55 | 0.5 | This work |  |
|  | *N_f_* | 6.00 x10^4^ | 1.20 x10^4^ | 1.90 x10^5^ | 5.40 x10^5^ | 2.56 x10^6^ | 1.00 x10^7^ |  |  |
| Cu-Cr | *r* | 0.7 | 0.65 | 0.6 | 0.55 | 0.5 | 0.45 | [7] |  |
|  | *N_f_* | 2.00 x10^5^ | 4.00 x10^5^ | 5.00 x10^5^ | 1.00 x10^6^ | 4.00 x10^6^ | 1.00 x10^7^ |  |  |
| Cu–Ag | *r* | 0.9 | 0.74 | 0.54 | 0.42 | 0.3 |  | [8] |  |
|  | *N_f_* | 1.00 x10^4^ | 2.00 x10^4^ | 4.00 x10^4^ | 2.00 x10^5^ | 1.00 x10^6^ |  |  |  |
| NT-Cu | *r* | 0.8 | 0.73 | 0.64 | 0.53 |  |  | [9] |  |
|  | *N_f_* | 6.00 x10^4^ | 2.00 x10^5^ | 1.00 x10^6^ | 1.00 x10^7^ |  |  |  |  |
| CG-Cu | *r* | 0.8 | 0.73 | 0.65 | 0.48 |  |  | [9] |  |
|  | *N_f_* | 1.20 x10^5^ | 2.50 x10^5^ | 1.00 x10^6^ | 1.00 x10^7^ |  |  |  |  |
| UFG-Cu | *r* | 0.8 | 0.7 | 0.5 | 0.4 | 0.3 |  | [10] |  |
|  | *N_f_* | 1.00 x10^4^ | 2.00 x10^4^ | 1.00 x10^5^ | 1.00 x10^6^ | 1.00 x10^7^ |  |  |  |
| NT-Cu-Al | *r* | 0.9 | 0.8 | 0.65 | 0.5 | 0.45 |  | [11] |  |
|  | *N_f_* | 1.00 x10^4^ | 3.00 x10^4^ | 2.00 x10^5^ | 2.00 x10^6^ | 4.00 x10^6^ |  |  |  |
| NT-Cu-Ni | *r* | 0.8 | 0.65 | 0.5 | 0.4 |  |  | [11] |  |
|  | *N_f_* | 2.00 x10^4^ | 1.00 x10^5^ | 1.00 x10^6^ | 4.00 x10^6^ |  |  |  |  |

**Table S2.** Comparison of average ratcheting strain rate and fatigue ratio for various metallic materials shown in Fig. 1e. For each material, the fatigue ratio *r*, average ratcheting strain rate $\dot{\varepsilon}_{r}/N$  (in s⁻¹), symbol and the source reference are listed.

| Materials |  |  |  |  |  |  |  | Ref. | Symbol |
| --- | --- | --- | --- | --- | --- | --- | --- | --- | --- |
| Cu-B_4_C ANP | *r* | 0.95 | 0.9 | 0.85 | 0.8 | 0.75 | 0.7 | This work |  |
|  | $\dot{\varepsilon}_{r}/N$ | 8.02 x10^-7^ | 7.59 x10^-7^ | 2.62 x10^-7^ | 9.49 x10^-8^ | 2.67 x10^-8^ | 4.50 x10^-10^ |  |  |
| Cu-B_4_C CNP | *r* | 0.9 | 0.8 | 0.7 | 0.6 | 0.55 |  | This work |  |
|  | $\dot{\varepsilon}_{r}/N$ | 7.93 x10^-5^ | 1.20 x10^-5^ | 4.77 x10^-7^ | 1.71 x10^-7^ | 2.73 x10^-8^ |  |  |  |
| UFG-Cu | *r* | 0.9 | 0.8 | 0.75 |  |  |  | [12] |  |
|  | $\dot{\varepsilon}_{r}/N$ | 1.60 x10^-5^ | 4.44 x10^-7^ | 2.82 x10^-7^ |  |  |  |  |  |
| GNG-Cu | *r* | 0.9 | 0.75 | 0.7 | 0.63 |  |  | [13] |  |
|  | $\dot{\varepsilon}_{r}/N$ | 1.56 x10^-5^ | 7.67 x10^-7^ | 6.40 x10^-8^ | 7.00 x10^-9^ |  |  |  |  |
| Cu-Nb | *r* | 0.95 | 0.925 | 0.9 | 0.85 |  |  | [14] |  |
|  | $\dot{\varepsilon}_{r}/N$ | 1.05 x10^-3^ | 1.80 x10^-4^ | 3.27 x10^-5^ | 1.05 x10^-5^ |  |  |  |  |
| 304 Steel | *r* | 0.9 | 0.85 | 0.7 |  |  |  | [15] |  |
|  | $\dot{\varepsilon}_{r}/N$ | 1.05 x10^-5^ | 2.58 x10^-6^ | 1.70 x10^-8^ |  |  |  |  |  |
| GDS 304 | *r* | 0.9 | 0.8 | 0.7 |  |  |  | [15] |  |
|  | *ε/N* | 2.00 x10^-6^ | 3.00 x10^-7^ | 5.50 x10^-9^ |  |  |  |  |  |
| UFG Ni | *r* | 0.85 | 0.8 | 0.75 |  |  |  | [16] |  |
|  | $\dot{\varepsilon}_{r}/N$ | 3.00 x10^-6^ | 1.00 x10^-6^ | 6.00 x10^-7^ |  |  |  |  |  |
| NC Ni | *r* | 0.85 | 0.8 |  |  |  |  | [16] |  |
|  | $\dot{\varepsilon}_{r}/N$ | 1.35 x10^-6^ | 2.00 x10^-6^ |  |  |  |  |  |  |
| UFG Al | *r* | 0.95 | 0.9 | 0.85 |  |  |  | [17] |  |
|  | $\dot{\varepsilon}_{r}/N$ | 1.70 x10^-4^ | 9.39 x10^-5^ | 2.57 x10^-5^ |  |  |  |  |  |
| Al-Fe_3_O_4_ | *r* | 0.8 | 0.78 | 0.74 |  |  |  | [18] |  |
|  | $\dot{\varepsilon}_{r}/N$ | 1.57 x10^-5^ | 7.30 x10^-6^ | 1.00 x10^-6^ |  |  |  |  |  |

**SI References**

[1]	Ungár, T., Borbély, A., "The effect of dislocation contrast on x‐ray line broadening: A new approach to line profile analysis," *Appl. Phys. Lett.* 69, no. 21 (1996): 3173, <https://doi.org/10.1063/1.117951>.

[2]	Wilkens, M., "The determination of density and distribution of dislocations in deformed single crystals from broadened X-ray diffraction profiles," *Phys. Status Solidi (a)* 2, no. 2 (1970): 359, <https://doi.org/https://doi.org/10.1002/pssa.19700020224>.

[3]	Dickson, J. I., Boutin, J., Handfield, L., "A comparison of two simple methods for measuring cyclic internal and effective stresses," *Mater. Sci. Eng.* 64, no. 1 (1984): L7, <https://doi.org/https://doi.org/10.1016/0025-5416(84)90083-1>.

[4]	Fournier, B., Sauzay, M., Caës, C., Noblecourt, M., Mottot, M., "Analysis of the hysteresis loops of a martensitic steel: Part I: Study of the influence of strain amplitude and temperature under pure fatigue loadings using an enhanced stress partitioning method," *Mater. Sci. Eng., A* 437, no. 2 (2006): 183, <https://doi.org/https://doi.org/10.1016/j.msea.2006.08.086>.

[5]	Handfield, L., Dickson, J. I., "The Cyclic Deformation of Titanium: Dislocation Substructures and Effective and Internal Stresses," in *Proceedings of the Defects, Fracture and Fatigue*: 37.

[6]	Yang, M., Pan, Y., Yuan, F., Zhu, Y., Wu, X., "Back stress strengthening and strain hardening in gradient structure," *Mater. Res. Lett.* 4, no. 3 (2016): 145, <https://doi.org/10.1080/21663831.2016.1153004>.

[7]	Masuda, C., Tanaka, Y., "Fatigue properties of Cu–Cr in situ composite," *Int. J. Fatigue* 28, no. 10 (2006): 1426, <https://doi.org/https://doi.org/10.1016/j.ijfatigue.2006.02.051>.

[8]	Freudenberger, J., Klauß, H. J., Heinze, K., Gaganov, A., Schaper, M., Schultz, L., "Fatigue of highly strengthened Cu–Ag alloys," *Int. J. Fatigue* 30, no. 3 (2008): 437, <https://doi.org/https://doi.org/10.1016/j.ijfatigue.2007.04.009>.

[9]	Pan, Q. S., Lu, Q. H., Lu, L., "Fatigue behavior of columnar-grained Cu with preferentially oriented nanoscale twins," *Acta Mater.* 61, no. 4 (2013): 1383, <https://doi.org/https://doi.org/10.1016/j.actamat.2012.11.015>.

[10]	Shute, C. J., Myers, B. D., Xie, S., Barbee, T. W., Hodge, A. M., Weertman, J. R., "Microstructural stability during cyclic loading of multilayer copper/copper samples with nanoscale twinning," *Scripta Mater.* 60, no. 12 (2009): 1073, <https://doi.org/https://doi.org/10.1016/j.scriptamat.2008.11.049>.

[11]	Heckman, N. M., Berwind, M. F., Eberl, C., Hodge, A. M., "Microstructural deformation in fatigued nanotwinned copper alloys," *Acta Mater.* 144(2018): 138, <https://doi.org/https://doi.org/10.1016/j.actamat.2017.10.057>.

[12]	Wu, Y., Yang, J., Shen, X., Zhu, R., "Cyclic Creep of Ultrafine-Grained Pure Cu Under Cyclic Tension Deformation," *J. Mater. Eng. Perform.* 26, no. 2 (2017): 837, <https://doi.org/10.1007/s11665-017-2500-6>.

[13]	Jing, L. J. 2020, "Fatigue property and cyclic deformation mechanism of gradient nanograined Cu." University of Science and Technology of China.

[14]	Sun, Q., Jiang, F., Deng, L., et al., "Uniaxial fatigue behavior of Cu–Nb micro-composite conductor, part I: Effect of peak stress and stress ratio," *Int. J. Fatigue* 91(2016): 275, <https://doi.org/https://doi.org/10.1016/j.ijfatigue.2015.08.025>.

[15]	Pan, Q., Ding, K., Guo, S., et al., "Superior resistance to cyclic creep in a gradient structured steel," *Science* 388, no. 6742 (2025): 82, <https://doi.org/10.1126/science.adt6666>.

[16]	Cheng, S., Xie, J., Stoica, A. D., et al., "Cyclic deformation of nanocrystalline and ultrafine-grained nickel," *Acta Mater.* 57, no. 4 (2009): 1272, <https://doi.org/https://doi.org/10.1016/j.actamat.2008.11.011>.

[17]	Karami, S., Piroozi, B., Borhani, E., "Fatigue-induced microstructure evolution and ratcheting behavior of ultrafine-grained (UFG) pure aluminum processed by accumulative roll bonding (ARB)," *Mater. Charact.* 196(2023): 112578, <https://doi.org/https://doi.org/10.1016/j.matchar.2022.112578>.

[18]	Karami, S., Borhani, E., Yousefieh, M., Karami, S., "The effect of ratcheting strain on post-ratcheting tensile test of metal matrix composites (MMCs) reinforced by Fe3O4 nanoparticles manufactured by the accumulative roll bonding (ARB) process," *Mater. Sci. Eng., A* 903(2024): 146691, <https://doi.org/https://doi.org/10.1016/j.msea.2024.146691>.
